# A Narrative Review of the Role of Estrogen (Receptors) in Melanoma

**DOI:** 10.3390/ijms25116251

**Published:** 2024-06-06

**Authors:** Diet Caerts, Maria Garmyn, Canan Güvenç

**Affiliations:** Department of Dermatology, University Hospitals Leuven, 3000 Leuven, Belgium; diet.caerts@student.kuleuven.be (D.C.); marjan.garmyn@uzleuven.be (M.G.)

**Keywords:** cutaneous melanoma, estrogen, estrogen receptor, oral contraceptives, hormone replacement therapy, pregnancy, parity, menarche, menopause

## Abstract

In this narrative review, we attempt to provide an overview of the evidence regarding the role of estrogen (receptors) in cutaneous melanoma (CM). We reviewed 68 studies and 4 systematic reviews and meta-analyses published from 2002 up to and including 2022. The prevailing presence of estrogen receptor β (ERβ) instead of estrogen receptor α (ERα) in CM is notable, with ERβ potentially playing a protective role and being less frequently detected in progressive cases. While men with CM generally experience a less favorable prognosis, this distinction may become negligible with advancing age. The role of oral contraceptives (OC) and hormone replacement therapy (HRT) in CM remains controversial. However, recent studies tend to associate the use of these exogenous hormones with a heightened risk of CM, mostly only when using estrogen therapy and not in combination with progesterone. On the contrary, the majority of studies find no substantial influence of in vitro fertilization (IVF) treatment on CM risk. Reproductive factors, including younger age at first childbirth, higher parity, and shorter reproductive life, show conflicting evidence, with some studies suggesting a lower CM risk. We suggest an important role for estrogens in CM. More research is needed, but the integration of estrogens and targeting the estrogen receptors in melanoma therapy holds promise for future developments in the field.

## 1. Introduction

The incidence of cutaneous melanoma (CM) is increasing worldwide, even in young adults [1]. The trend is also evident in Belgium, with rising annual rates [2]. In most countries, CM is more prevalent in men than in women over 50 years old [1]. There is also a significant difference in prognosis between men and women, which cannot be completely explained by behavioral variations (e.g., women are more likely to seek health care and practice healthy behaviors). A more plausible explanation lies in the differences in immune systems and estrogen levels and their role in immunomodulation [3].

Güvenç et al. already highlighted the importance of the estrogen receptor pathway in their investigation. They observed a notable upregulation of the estrogen receptor pathway in thick, non-metastasizing CM. This upregulation was specifically identified in key members of the cytochrome P450 family, including CYP1A1, CYP1A2, and CYP1B1, along with an increased expression of estrogen receptor alpha (ERα). Moreover, the estrogen receptor pathway emerged as a significant finding and occupied a leading position in the list of identified pathways [4]. In line with this, other researchers discovered a better therapy response and a higher survival rate in women, who have higher estrogen levels than men. Interestingly, the same was found in obese men. The higher adipose tissue aromatase activity converts androgens to estrogen compounds, which in turn results in higher estradiol levels [5,6,7,8]. A possible mechanism of estrogen signaling is suggested by Smalley et al. In women with CM, estrogen activates GPER1. This induces increased signaling through the kinase PKA and the transcription factor CREB. In this way, there is a higher manifestation of the transcription factor MITF. Finally, this activates pigmentation genes. Simultaneously, PKA suppresses the transcription factor c-Myc. In this way, expression of the cell-surface protein HLA is no longer inhibited, and ultimately, the immune system (T-cells) increasingly recognizes the CM. In contrast, male CM patients have less estrogen. As a result, GPER is not activated, and pigmentation genes are not as active. The high levels of c-Myc inhibit the production of HLA and activate the immune checkpoint inhibitor PD-L1 [9]. In breast cancer, ERα downregulates miR-140, which targets transcription factor SOX2, and in this way regulates breast tumor-initiating cells [10]. This role can be hypothesized in CM as well, but it is still under debate [11,12]. Following this ongoing evidence, estrogens and estrogen receptors (ERs) have a potential and important role in CM outcomes. It could provide valuable insights for both understanding CM pathobiology and developing targeted therapies.

If there is a substantial difference between sexes, this may also be the case between women. A woman goes through different life stages, all with different estrogen levels (e.g., childhood, puberty, pregnancy, menopause, etc.) [13]. One noteworthy life stage is pregnancy, when more sex hormones circulate, which could possibly affect the CM outcome. However, it is also possible that the physician focuses on the (physiological) increased skin pigmentation during pregnancy, which distracts him or her from changing naevi [14]. Of course, exogenous hormones, such as oral contraceptives (OC) in the reproductive phase or hormonal replacement therapy (HRT) in postmenopausal women, have become ubiquitous in our lives and may also have an impact [15]. The World Health Organization claims that estrogens are carcinogenic, but Ruan et al. do not agree with this and state that they can even have cancer-protective effects [16].

Estrogens bind to estrogen receptors such as ERα and estrogen receptor beta (ERβ), both of which belong to the group of nuclear receptors. There is also a third receptor: the G-protein-coupled estrogen receptor 30 (GPR30 or GPER1). This is a membrane estrogen receptor. There are various modes of ER signaling (direct-, indirect genomic-, and indirect non-genomic signaling) via different classes of ligands (endoestrogens, phytoestrogens, xenoestrogens, metalloestrogens, and selective estrogen receptor modulators (SERMs)) or via ligand-independent pathways. SERMs can act in different tissues as ER agonists and antagonists (e.g., Tamoxifen). In addition, there are aromatase inhibitors that block estrogen signaling [17,18,19]. For example, Ohata et al. and Rajabi et al. found that normal skin contained mainly ERα, as opposed to mainly ERβ in CM [20,21]. Among others, Bhari et al. hypothesized that ERβ expression decreased with increasing Breslow thickness. They also suggested that women have more ERβ than men and thus a better prognosis with less aggressive and less metastasizing forms of CM [22]. In general, ERβ has a tumor-suppressive effect and can thus potentially be implemented in therapy [23].

In this review, our primary focus will be on the estrogenic aspect of CM cases. By concentrating on the estrogenic components, we aim to unravel the molecular intricacies of CM populations. By exploring the different estrogenic components (estrogen (receptors), the difference between sexes, exogenous estrogens, and the different life stages) and their associated mechanisms, we aim to gain a profound understanding of the role of estrogen in the pathobiology of cutaneous melanoma (CM).

## 2. Results

### 2.1. Estrogen (Receptors)

Our findings regarding estrogen and estrogen receptors (ER) are summarized in Table 1. First, we review the studies related to estrogen receptor alpha (ERα). Mori et al. frequently detected methylated ERα in CM tissue. Because of these observations, they evaluated the presence of methylated ERα in the serum of stage I to IV CM patients and its role as a disease detection marker. Serum hypermethylated ERα was less detected in localized compared to advanced CM, and it was unfavorably prognostic for the outcome of CM and response to therapy [24]. Glatthaar et al. evaluated four ESR1 (gene for encoding ERα) single-nucleotide polymorphisms (SNPs) in blood samples: +2464C/T, −4576A/C, +1619A/G, and +6362C/T. When the SNP 6362C/T was located on the variant allele, significantly more patients had a disease course of less than a year and fewer relapses. There were no overall differences if it was located on the wild-type allele or heterozygote. Patients with SNP −4576A/C on the wild-type allele had a decreased risk of disease development but needed significantly more chemotherapy compared to when localized on the variant allele. Cases carried significantly more often the +1619A/G wild-type allele, and controls carried significantly more often the +1619A/G and +6362C/T variant alleles [25]. In contrast, Rajabi et al. did not find any ERα in CM tissue using immunohistochemistry [21].

On the other hand, there is estrogen receptor beta (ERβ). De Giorgi et al. showed that ERβ expression is significantly lower in CM compared to healthy skin and in thick compared with thin CM. They mentioned an inverse relationship between Breslow thickness and ERβ expression. According to these results, ERβ could be a positive prognostic factor for CM outcome [26]. In line with this, Asadi et al. reported lower ERβ protein levels in CM tissue. They suggested the use of ERβ to distinguish between malignant and benign lesions [27].

Other studies looked simultaneously at ERα, Erβ, and other ERs. Schmidt et al. stated that ERβ, and not ERα, is predominantly present in all melanocytic lesion types. The strongest ERβ immunostaining was seen in melanocytes in dysplastic nevi with profound cytological atypia and in lentigo malignas. The ERβ levels were correlated with the tumor microenvironment (e.g., melanocytes close to keratinocytes > Clark Level III/IV or thick CM (Breslow)). ERβ immunoreactivity decreased with increasing Breslow depth; with this in mind, they suggested that ERβ loss can reflect a CM stage in which it becomes independent of estrogen [28]. In line with these results, Ohata et al. did not find any ERα expression in CM and melanocytic nevi, unlike Erβ, which was present [20]. The study by De Giorgi et al. detected ERα and ERβ mRNA as well as ERβ protein in all melanocytic lesions. They found that as CM progresses, the expression of both ERs decreases. They identify ERβ as a potentially favorable prognostic factor [29]. De Giorgi et al. suggested that an ERβ polymorphism at the AluI restriction site correlated with a higher CM susceptibility, and the pp polymorphism showed a significant difference in Breslow thickness. They also suggested that polymorphisms in ERα and ERβ may negatively influence tumor progression [30]. Spyropoulos et al. most prevalently detected ERβ, and not ERα, in all primary CM and sentinel lymph nodes. Negative sentinel lymph nodes associated with thinner, less invasive CM had the most intense ERβ immunostaining. Thus, there is less ERβ expression in aggressive CM with sentinel nodal metastatic disease, indicating its possible use as a surrogate marker for CM prognosis [31]. In addition, Fabian et al. saw a lower Breslow thickness, a lower mitotic rate, and a higher presence of peritumoral lymphocyte infiltration when CM was ERβ-/GPER-positive (G-protein-coupled estrogen receptor). GPER was associated with a thinner CM, a lower mitotic rate, and fewer metastases. ERβ positivity was more common in thinner CM and inversely associated with aggressive, sentinel lymph node-positive CM, mitotic rate, and Breslow depth. In the majority of GPER-positive CM, ERβ was also present. GPER co-expressed with ERβ had a superior CM outcome. This double ER expression may be a novel prognostic indicator [32].

Yuan et al. selected different SNPs in ESR1, ESR2 (the gene for encoding ERβ), insulin-like growth factor 1 (IGF1), and insulin-like growth factor 1 receptor (IGF1R). Estrogen can induce the expression of IGF1R and, in this way, initiate the IGF1 signaling pathway and thus cell proliferation. IGF1 and IGF1R SNPs may provide information on the ER/IGF1R network in CM. In the international Genes, Environment, and Melanoma study (GEM) dataset, the variants of rs1520220 in IGF1 and rs2229765 in IGF1R were significantly related to CM risk, but not in the Gene Environment Association Studies Initiative (GENEVA) dataset. Further analysis showed that the GG phenotype in IGF1 rs1520220 showed an increased male CM risk but a significant contrary effect in women. The AA genotype in IGF1R rs2229765 showed a profound male protective effect, but not in women. They also suggest a role for the ESR1 SNPs rs2234693 and rs827421 in CM patients [33].

The study by Spalkowska et al. reported higher ERβ expression in dysplastic nevi margin melanocytes compared to common nevi, in dysplastic nevi keratinocytes compared to CM keratinocytes, and significantly higher in margin melanocytes compared to CM melanocytes. GPER expression was higher in dysplastic nevi, at the margin, or in skin margin tissue melanocytes compared to CM or CM melanocytes. GPER was also higher in the sebaceous gland surrounding dysplastic nevi compared to common nevi. There were no different ERα expressions between the melanocytic lesions [34]. Larsson et al. did not find a strong association between the endogenous hormone 17β-estradiol (E2) in serum and cancers such as CM in women [35]. Lastly, Dika et al. detected nuclear ERβ in all CM cases of female patients with a breast cancer history and cytoplasmatic ERβ in a few. This ERβ expression was much lower in the control group. Almost all CM cases of female patients with a history of breast cancer contained cytoplasmic ERα. Female CM cases treated with anti-estrogen therapy were overall more likely to display ERs compared to women of similar age and CM staging and women of childbearing age, both with and without a history of ovarian stimulation [36].

### 2.2. Differences between Sexes

Table 2 compiles studies that investigated sex differences; some of them observed a positive prognosis in females. De Vries et al. found a female superior survival that persisted after adjusting for multiple confounders, such as age, Breslow thickness, histological type, stage, and body site. Men more often had nodular CM, CM localized on the trunk and head or neck, higher Breslow thickness, and nodal or visceral involvement [37]. In line with this, Joosse et al. demonstrated an overall better survival rate for women with CM than men. This manifested itself in a lower progression risk and risk of lymph node or visceral metastasis and a survival advantage after first progression and metastasis in women. Lentigo maligna CM and acral lentiginous CM were more prevalent in women. Local recurrence, in-transit metastases, and the incidence of superficial spreading CM (SSM) and nodular CM were equally common in both sexes [7]. In addition, Joosse et al. found a female advantage in overall and disease-specific survival, time to lymph node, and distant metastasis in stages I and II CM. This female advantage was possibly not the case with head and neck CM [38]. Furthermore, Joosse et al. looked at stages III and IV CM, and again, there was a consistent female advantage. Interestingly, the advantage seemed smaller with a higher metastatic tumor load. They ruled out behavioral variations as an explanation [39]. Similarly, Morgese et al. revealed that women with stage I and II CM had a survival benefit compared to their male counterparts, but no difference was seen in stages III and IV [40].

Other studies noted a worse prognosis in men, as did Scoggins et al. They exhibited a greater incidence of negative CM characteristics in men without an increased risk for nodal metastasis. They highlighted sex as an independent survival factor [41]. In line with this, Gamba et al. reported worse male survival among adolescents and young adults with CM. They suggested a biological rather than a behavioral explanation [42].

Some studies discussed the outcomes for both sexes. In the study of Strouse et al., men had a lower CM incidence but, at the same time, an unfavorable prognosis, as did older participants with CM. Cases occurred earlier in women [43]. Lasithiotakis et al. mentioned that both old age (more obvious in women) and male sex were prognostically unfavorable. Women had a better prognosis, but this was no longer statistically significant above 65 years old [44]. Enninga et al. demonstrated an increased relative mortality risk with older age, more pronounced among women. Women with localized and regional CM had a decreased mortality risk compared to men within all age groups. This was mostly when CM was located on the face, ears, trunk, or extremities, but not on the scalp or neck. In patients with metastatic CM, there was no survival difference between the sexes and no age effect. This indicated that the role of sex in survival is restricted to early-stage disease [45].

In the study of Liu et al., women aged 44 years or younger showed higher CM incidences, in contrast with older men, who showed higher incidences. Exposure to solar ultraviolet (UV) radiation was the major cause of CM at an older age, but they suggested the role of other factors, such as hormones. They may play a role in early-onset CM, particularly in women [46]. Following the study of Hernando et al., it is possible that higher estrogen levels in women cause differentially expressed melanogenic SNPs, which might cause darker pigmentation or sun protection. These sex-specific genetic effects could also explain the higher CM risk in men. The following SNPs possibly showed a strong difference in CM risk: TYR, SILV/CDK2, GPR143, and F2RL1 [47].

Schmidt et al. discovered more ERβ immunoreactivity in female non-CM melanocytic lesions compared to male lesions, although this was not statistically significant. In the same way, they could not reveal a significant difference in ERβ immunoreactivity in CM in both sexes [28]. In the same way, Ohata et al. could not find a sex difference in ERβ immunostaining in melanocytic lesions [20]. De Giorgi et al. reported a more pronounced ERβ loss in CM compared with adjacent healthy skin in men and in postmenopausal women compared to premenopausal women. This may explain the female survival benefit [26]. Fabian et al. did not find a difference in GPER/ERβ expression in CM lesions between both sexes [32].

### 2.3. Exogenous Estrogens

The studies summarized in Table 3 discuss exogenous estrogens. First, there is oral contraceptive (OC) use. Vessey et al. did not find a correlation between CM and OC use or interval since last use, and also no correlation with a diaphragm or an intrauterine device [48]. By contrast, Benyi et al. suggested an increased CM risk with high-dose estrogen treatment for tall women during adolescence. Because of the small number of CM cases, they do not rule out the possibility that their findings are coincidental [49]. In line with this, Cervenka et al. concluded that long-term and high-dose users had a strong association with CM. There was an inverse association with age at first OC use and no association with age at or time since last use. A positive association was found between OC use and tanning bed use, sunburns, and sunscreen use. Because of these possible confounders, their findings only supported a weak association between OCs and CM risk [50].

Other studies focused on hormone replacement therapy (HRT). Mackie et al. did not find an adverse prognosis for CM with HRT after stage I or II CM surgery. They suggested that such therapy may even improve the prognosis, i.e., a lower proportion of ulceration and no difference in CM thickness [51]. In the same way, Tang et al. did not find an effect of HRT (estrogen alone or combined with progesterone) on the CM incidences. No statistically significant different subtypes of CM (invasive vs. in situ) were obtained between women randomly attributed to estrogen alone vs. placebo, estrogen-progesterone combined vs. placebo, or any hormone therapy vs. placebo [52]. On the contrary, Simin et al. found a limited increase in CM with HRT, but there was no difference between estrogen or estrogen combined with progesterone [53]. In line with these findings, Botteri et al. reported an increased CM risk with the current use of HRT estrogens (oral and vaginal); this was not observed in combinations with progesterone. Higher oral doses of estrogens increased the CM risk, and it decreased with higher progesterone doses. They suggested an opposite effect of estrogen and progesterone [54]. In the same way, Cervenka et al. demonstrated a moderately higher CM risk with HRT, mostly lentigo maligna CM and CM of the head and neck. The strongest association was seen between past users and users of norpregnane derivatives (not significant). The risk was similar across different durations of use and HRT types. Treatment onset shortly after menopause (less than six months) increased the risk. HRT users reported more sunscreen use than non-users [55]. Hicks et al. showed that high HRT use was associated with a moderately higher CM risk without evidence of a dose-response pattern. This was most pronounced amongst recent high users, for localized disease and for intravaginal estrogen therapy. There was no association between secondary CM and post-diagnostic new-use or continuous HRT use compared with non-use. No evidence about the CM prognosis was found. These scientists pointed out a possible detection bias in their study because of the more frequent health care contacts among HRT users [56]. Botteri et al. reported that HRT use, especially estrogen, was associated with an increased CM risk, but this was not dose-dependent. There was no association with estrogen-progesterone therapy use for less than 5 years [57]. In the systematic review and meta-analysis of Tang et al., estrogen HRT gave a higher CM risk. This was less high when progesterone was combined. The association between HRT and CM positively increased in the first 3 years of usage. After that, the response flattened and was reduced [58]. Botteri et al. indicated a possible association between CM risk and estrogen therapy, but not with estrogen-progesterone therapy [59]. Lallas et al. concluded in their systematic review and meta-analysis that women who currently took estrogen and estrogen-progesterone HRT had a higher CM risk, more specifically SSM and lentigo maligna CM, but not nodular CM [60].

Several studies examined the use of both OC and HRT. Freedman et al. found no association between these exogenous hormones and CM, although there was a non-significantly elevated risk with OC use [61]. Similarly, Naldi et al. and Lea et al. also reported no role of OC or HRT in CM risk [62,63]. The systematic review and meta-analysis of Gandini et al. did not find an effect of OC or HRT on CM. They investigated different aspects, such as ever use, duration of use, age at first use, time since last use, etc. [64]. Donley et al. did not find an association between OC or HRT use and CM risk, even when the duration of use was considered [65]. Further, Koomen et al. could not conclude an association between OC and HRT use and Breslow thickness [66].

In contrast, Koomen et al. suggested a cumulative dose-dependent increase in CM risk associated with the use of estrogens in OC and HRT [67]. Interestingly, De Giorgi et al. revealed that women using an OC or HRT had a lower CM risk than men. This contrasted with nonusers, who had an increased risk compared to men [68]. According to Cervenka et al., women who used OCs at any time had a borderline significantly increased risk of CM, increasing with prolonged use. HRT non-significantly increased the CM risk; furthermore, there was no association with duration of use or age at first intake. The researchers concluded that there was only a weak association between exogenous hormones and CM risk. SSM was detected most frequently [69]. Sun et al. showed in their systematic review and meta-analysis that women with OC use for more than five years and first use fifteen to nineteen years prior had an increased CM risk. They found no effect of years since last use or age at first use. HRT also increased the risk, especially for SSM, but not for nodular CM. They pointed out estrogen and estradiol as the most active causative agents in CM risk and not progesterone, although they did not rule out the role of sun exposure [70].

Other exogenous hormones were also examined. According to Althuis et al., there was no significantly increased CM risk with clomiphene or gonadotrophin use. However, its use may pose stronger risks among nulliparous women [71]. The study by Hannibal et al. demonstrated no robust association between CM risk and the use of fertility drugs such as clomiphene, gonadotrophins, human chorionic gonadotrophin (hCG), or gonadotrophin-releasing hormone (GnRH). Nevertheless, gonadotrophins or GnRH might elevate the risk in women who have had children. The study considered ever use, the number of cycles used, and years since the first use [72]. Silva et al. did not observe an association between ovarian stimulation, such as clomiphene and gonadotrophins, and CM [73]. Yli-Kuha et al. did not find a statistically significant increase in general cancer risk, including CM, with in vitro fertilization (IVF) drugs [74]. Spaan et al. reported no higher CM risk among women who were treated with IVF drugs [75]. Mai et al. did not find an association between CM risk and OC or HRT use, or the DES taken by the mother [76].

Calderon-Margalit et al. found a significantly increased CM risk when treated with clomiphene, in contrast to other ovulation induction therapies. This association was stronger when it took longer than one year to become pregnant [77]. In the study of Verloop et al., women exposed to diethylstilbestrol (DES) in utero were investigated. They appeared to have a significantly increased risk for CM diagnosis before the age of 40 [78]. Stewart et al. detected an increased rate of CM in women who had IVF and gave birth, compared with women who had IVF and did not give birth. Moreover, women who had non-IVF infertility treatment and gave birth did not have an increased rate of CM. They suggested a possible association between reproductive factors and CM in women who undergo IVF treatment [79]. Brinton et al. observed a significantly higher CM risk with every use of clomiphene, primarily in women who used lower dosages [80]. Lai et al. investigated exogenous hormones, such as phytoestrogens, in food. They found that patients with a CM history were less likely to consume tofu and thus had lower urinary phytoestrogen levels. Although this correlation was not statistically significant, no further conclusions could be drawn [81]. Dika et al. showed cytoplasmic ERα and PR positivity in CM occurring after IVF but not during pregnancy, not in atypical naevi during pregnancy, and not in atypical naevi during pregnancy who had undergone IVF. They could not rule out the possibility that this was a sporadic phenomenon [82]. Finally, Dika et al. detected an overall lower abundance of nuclear ERs in CM in the group of female patients undergoing ovarian stimulation [36].

### 2.4. Different Life Stages

Hormone levels undergo fluctuations throughout a woman’s life, with pregnancy representing a crucial life stage. Our findings in this regard are succinctly presented in Table 4. Daryanani et al. discerned that pregnancy did not exert an adverse, enduring impact on the survival of patients diagnosed with localized stage I or II CM when compared to their non-pregnant counterparts. Notably, pregnant women did not exhibit statistically significant differences in tumor thickness or histological subtype, with the majority being SSM. Instead, the prognosis still hinged on variables such as tumor thickness and ulceration [83]. In line with this, Lens et al. reported no significant difference in Breslow thickness or survival between pregnant and non-pregnant women with CM. Pregnancy following the diagnosis of primary CM was not associated with an increased mortality risk, nor was pregnancy associated with new CM development or the recurrence of a previously treated CM. Again, prognosis depended on Breslow thickness, and head, neck, or trunk tumors had a worse prognosis [84]. The study by O’Meara et al. demonstrated an excellent outcome for pregnant women with localized CM in different aspects, such as tumor thickness, stage, lymph node metastasis, and survival. In essence, their outcome was equivalent to that of pregnant women without CM [85]. Hannibal et al. did not find a significant association between parity, number of live births, age at first birth, age at last birth, and CM. The risk seemed slightly lower among parous women compared to nulliparous women [72].

Neale et al. found a positive association between CM risk and older age at first childbirth and a significant inverse association with the number of maternities. Mothers of twins and singletons had the same risk [86]. Similarly, Karagas et al. highlighted a lower CM risk in women with a younger age at first birth and higher parity; when these factors were combined, the effect was even stronger. There was no overall association with ever having a live birth [87]. Kaae et al. reported a significantly lower CM risk among high-parity women. Women older than 25 at their first birth had a higher CM risk than younger women. Ten or more years after her first birth, a woman had a higher CM risk than she did in the first years. Surprisingly, the similarity of observations between both sexes suggested that lifestyle factors, rather than exposure to pregnancy hormones, might be responsible [88]. The systematic review and meta-analysis of Gandini et al. found a significantly increased risk with older age at first pregnancy and a significantly lower risk with higher parity [64]. Spaan et al. reported that women with a younger age at first birth (<30 years) had a significantly lower CM risk. Nulliparous women did not have a significantly increased CM risk compared to parous women [75]. The results of Miller et al. showed a negative effect of pregnancy on the CM course, with significantly more metastatic sentinel nodes and borderline significantly higher mortality. The pregnant group also had a non-significantly higher mean Breslow thickness [89].

Several years later, Zhou et al. demonstrated a higher expression of ERβ in the CM of pregnant women than in the CM of men, without a difference between pregnant and nonpregnant women. However, these findings were not statistically significant. There was no link between ERβ expression and survival, Breslow thickness, primary tumor, metastasis, or disease stage. None of the cases expressed the androgen receptor, and only two cases expressed ERα [90]. Fabian et al. showed a significantly higher GPER and ERβ presence in the CM of pregnant women than in the CM of non-pregnant women or men. In the non-pregnancy-associated CM, only ERβ is frequently found. In pregnant patients, GPER positivity was inversely associated with tumor ulceration. There was no survival difference between pregnancy- or non-pregnancy-associated CM [32]. Hrgovic et al. observed significantly elevated levels of serum 2-methoxyestradiol (2-ME) in pregnant control patients compared with CM patients and non-pregnant healthy controls. There was no association between 2-ME serum levels and CM disease stage, tumor thickness, lactate dehydrogenase, or S100 calcium-binding protein B levels. The serum levels were not decreased in CM patients and did not differ significantly from those of normal healthy controls. Therefore, endogenous serum 2-ME levels did not seem appropriate as a prognostic value in CM, in contrast to the potential therapeutic effects of exogenous 2-ME [91].

The investigation into hormone levels extends to pivotal life stages such as menarche, menopause, and old age. Various studies have delved into these stages, with some also exploring the nuances of pregnancy. Freedman et al. contributed to this exploration and reported that there is no discernible impact of age at menarche, menopausal status, parity, or age at first birth on the aspects under consideration [61].

Naldi et al. found no association between CM and the number of live births, the number of abortions, menstrual factors, or hormonal factors, including menopause. Late age at first and last birth were significantly associated with a risk of CM [62]. In the study of Lea et al., there was an association between live birth five years before diagnosis and between the number of births and CM risk in women under 55 years. There were no differences between cases and controls by age at menarche or menopause or age at first or last pregnancy [63]. Kvaskoff et al. identified a reduced CM risk in women aged fifteen years or older at menarche, younger than 48 years at menopause, with irregular menstrual cycles, or with shorter reproductive or menstrual lives. They found a modest decreased risk with higher rates of pregnancies, parity, and history of miscarriages. They found no association between age at which menstruation was regular, age at first or last birth, time since last birth, breastfeeding duration, or menopausal status, and CM risk. After taking everything into consideration, they suggested that decreased exposure to ovarian hormones caused a reduced CM risk [92]. Following Donley et al., an increased CM risk was associated with young age at menarche and older age at menopause. Parity and age at first birth were not associated with CM risk. They hypothesized that endogenous estrogen exposure in childhood increases photocarcinogenicity [65]. Sun et al. concluded in their systematic review and meta-analysis that women with a parity of three or more births had a reduced CM risk. This was in contrast to women who gave birth for the first time when they were 20 years of age or older; they had an increased CM risk compared with younger women. They did not find an association with age at menarche or menopausal status [70]. According to Mai et al., participants with older age, menarche at an earlier age, and older age at first birth had a higher CM incidence. No significant association was found for other reproductive factors [76].

As previously mentioned, Joosse et al. found an overall better prognosis for CM in women compared to men. The female advantage compared to men of the same age was equal in the postmenopausal and premenopausal groups. Only the disease specific survival advantage was no longer significant in the postmenopausal group [38]. Joosse et al. looked at stages III and IV of CM. Again, there was a consistent female advantage, also in pre- and postmenopausal groups [39]. Enninga et al. found a better prognosis in women in localized and regional CM, both in pre- and perimenopausal age [45].

Ohata et al. could not find a difference in ERβ immunostaining in melanocytic lesions of different age groups or between pregnant and non-pregnant patients [20]. De Giorgi et al. found a more pronounced ERβ loss in CM compared with adjacent healthy skin in postmenopausal women compared with premenopausal women [26].

## 3. Discussion

In our attempt to offer a comprehensive overview of the literature regarding the role of estrogen and estrogen receptors in melanoma, we incorporated findings from a total of 68 studies, along with four systematic reviews and meta-analyses.

### 3.1. Estrogen (Receptors)

Our investigation revealed that predominantly estrogen receptor beta (ERβ) and not estrogen receptor alpha (ERα) are present in cutaneous melanoma (CM) [20,21,28,31]. ERβ may have a protective role and is less prevalent in progressive CM [24,26,27,28,29,31,32,34]. Consistent with our findings, an animal study conducted by Cho et al. demonstrated that, specifically, ERβ signaling has a natural endogenous protective role against CM growth [93]. Contradictory, Larsson et al. did not find a significant role for estradiol, possibly challenging these assumptions [35]. There are contrasting findings regarding the presence or absence of ERα in CM tissue and its role in CM progression. While some suggest a negative prognostic role, others could not detect ERα in CM tissue or found less ERα in progressive CM [20,21,29]. Our investigation also identified a role in CM risk associated with certain receptor polymorphisms, which underscores the complex interplay of estrogen receptors and genetic factors in CM [25,29,30,33].

ER is also relevant in breast cancer. Hashmi et al. investigated ER and PR (progesterone receptors) in metaplastic breast carcinoma. These ER and PR positive cancers had a higher recurrence rate, were smaller, and were of a lower grade and stage than the ER- and PR-negative variants. Furthermore, though, no significant survival advantage was found [94].

### 3.2. Differences between Sexes

When it comes to early-stage disease, numerous studies have reported a more favorable prognosis in women. Nevertheless, this difference in prognosis between the two sexes became less pronounced with age. Hormonal changes related to menopause have been proposed as a potential explanation for these findings [7,37,38,39,40,41,42,43,44,45]. In line with this, De Giorgi et al. identified lower levels of ERβ in men and postmenopausal women [26]. However, other studies did not find significant differences in ERβ levels between men and women with CM [20,28,32].

### 3.3. Exogenous Estrogens

The majority of studies consistently suggest an increased risk of cutaneous melanoma (CM) associated with oral contraceptives (OC) and hormone replacement therapy (HRT), particularly estrogen therapy alone, possibly due to progesterone’s counteractive effects on estrogen’s stimulation [49,50,53,54,55,56,57,58,59,60,67,70,77,78,79,80]. However, conflicting findings exist, with some studies failing to establish a clear association, potentially influenced by outdated data and changes in hormone formulations over time [48,64,65]. Notably, the study by de Giorgi et al. even suggested a lower CM risk among women using OC or HRT and attributed this to the predominance of ERβ in melanocytes. However, careful interpretation is required because the controls were not adequately matched with cases and potential confounding factors, such as sun exposure, were not thoroughly controlled for [68]. In exploring the role of exogenous hormones, two letters to the editor contributed additional valuable insights. Mueller et al. reported no significant association between estrogens and CM, attributing this to the lower doses of estrogens currently in use [95]. Similarly, Olsen et al. observed only a modest association between long-term oral OC use and invasive CM [96].

Most studies investigating the role of various in vitro fertilization (IVF) drugs did not identify a significant association with CM [71,72,73,74,75,76]. However, there are limitations to consider. For instance, Hannibal et al. noted the diverse array of drugs used by participants, while Yli-Kuha et al.‘s study had a small number of CM cases, lacked subgroups, and provided limited information about different drugs and dosages [72,73]. In contrast, other researchers did find a higher risk with clomiphene use. Remarkably, Brinton et al. primarily reported this with low dosages, raising questions about the biological plausibility of this relationship [80].

The exploration of diet, especially the role of phytoestrogens, has not been extensively addressed in our research. Lai et al. suggested the potential protective role of a tofu-based diet, but these findings did not reach statistical significance [81]. Similarly, in their animal study, Tian et al. proposed a link between a high-cholesterol diet and CM progression, potentially mediated by ERα [97].

These perspectives emphasize the nuanced relationship between exogenous hormones and CM risk. They underline the need for rigorous research on the evolution of current drug exposure, compounds, and usage patterns. Also, future research on diet and specific components, such as phytoestrogens, could provide valuable insights for developing preventive and therapeutic strategies for CM. This in-depth research can be accomplished in the format of prospective cohort studies where estrogen use and dietary habits of the population can be examined.

### 3.4. Different Life Stages

The reported relationship between reproductive factors and CM risk has been ambiguous. Most researchers indicated a lower CM risk with a younger age at first childbirth and higher parity [62,64,70,75,76,86,87,88]. Remarkably, given the similar results in men, Kaae et al. suggested the possible influence of lifestyle factors [88]. Additionally, Miller et al. reported a worse prognosis in pregnancy. However, their results were based on a small sample size, so no reliable conclusions can be drawn [89].

Results related to CM risk and the age of menarche or menopause are also inconclusive. While some studies suggest a higher risk with a young age at menarche and an older age at menopause, others, like Olsen et al., found no significant role at all [96]. Additionally, Joosse et al. indicated an equally good prognosis of CM in pre- and postmenopausal women compared to men, while Enninga et al. concluded a better CM prognosis in pre- and perimenopausal women compared to men. However, caution is warranted as these studies drew conclusions without concrete information about menopausal status and instead divided women into menopausal categories based on age [38,39,45].

In line with this, the exploration of hormone receptors in CM during different life stages has yielded mixed findings. Some studies reported elevated ERβ and GPER levels in CM during pregnancy, along with increased levels of 2-methoxyestradiol (2-ME), without a discernible effect on CM [32,91]. Other studies, in turn, found no difference in ERβ immunostaining between pregnant and non-pregnant women [90]. Postmenopausal women, compared with premenopausal women, may have had a more pronounced loss of ERβ in CM [26]. The complex interplay of reproductive factors and CM risk underscores the need for ongoing research to unravel the intricacies of this relationship. Again, the use of prospective cohort studies could be helpful in this regard for addressing the identified gaps.

### 3.5. Implementation in Therapy

This narrative review highlights potential therapeutic and preventive strategies for CM involving estrogens. In breast cancer research, it is already long known that ERα is a possible therapeutic target in more than 70% of breast cancers. ERα is antagonized by selective estrogen receptor modulators or degraders. Because of hormonal resistance, dual target inhibitors are also seen as a possibility [98].

Several animal studies provide insights into these possibilities. Zhao et al. suggested ERβ activation to suppress lung metastasis in CM [99]. Yuan et al. proposed the implementation of ERβ agonists in combination with immune checkpoint blockade immunotherapy [33]. Similarly, Wu et al. demonstrated the potential use of acteoside in regulating the ERβ-Ras/Raf1-STAT3 signaling axis, inducing tumor cell apoptosis, and inhibiting the occurrence and development of CM [100]. Péqueux et al. discussed the potential promoting role of estradiol in certain cancer cells, while Dobos et al. and Hua et al. suggested anti-CM effects of 2-ME, with the latter proposing its combination with PD-1 blockade [101,102,103]. Li et al. demonstrated tumor suppression by injecting estradiol, and Natale et al. showcased a collaboration between GPER and immune checkpoint blockade [104,105]. The only human study mentioned is by Bechman et al., suggesting targeting ERβ together with nitric oxide release to reduce tumor growth and prevent metastasis [106].

## 4. Materials and Methods

### 4.1. Search Strategy

We searched the electronic databases PubMed, EMBASE, ClinicalTrials.gov, and Trialsearch.who.int on 30 December 2022, for eligible studies. The used search terms and concepts are listed in Table A1. These concepts were expressed in Mesh-terms (PubMed), Emtree-terms (Embase), or free text.

### 4.2. Study Selection

**Figure 1:**
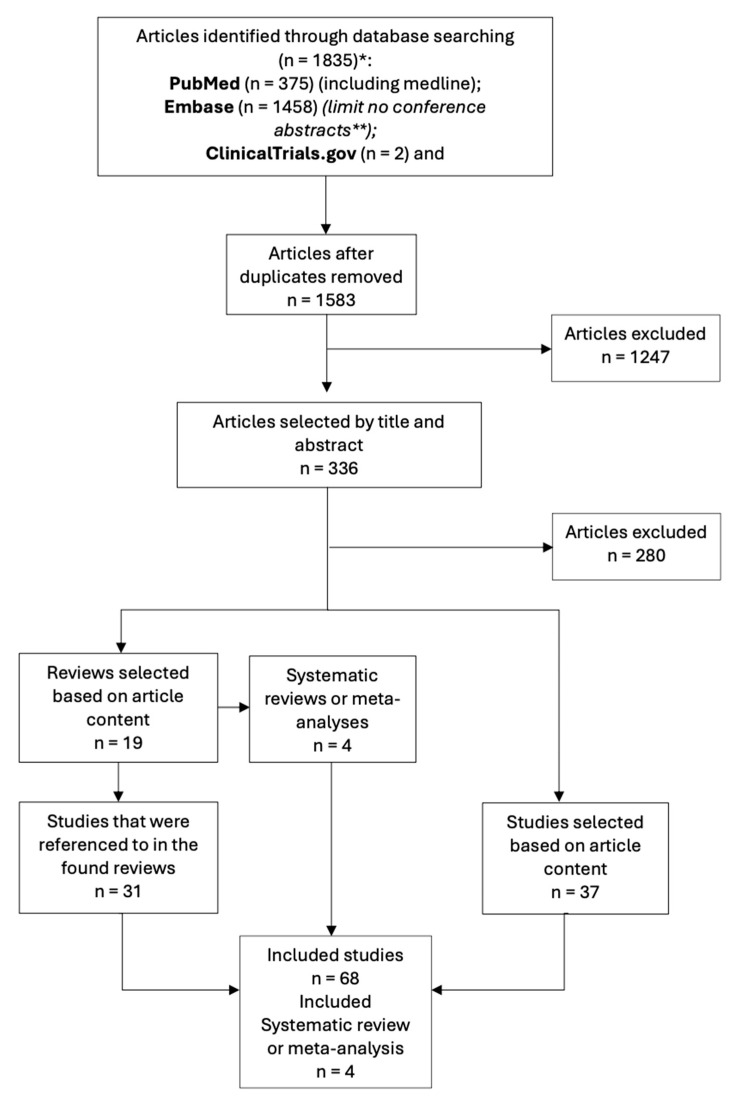
Flow diagram according to The Preferred Reporting Items for Systematic Reviews and Meta-Analysis [107] with the detailed search process on 30 December 2022. * Time limit from 2002 up to and including 2022 ** No conference abstracts were used because they are of no use in this narrative review. Remarks: no published search filters were used.

The study selection process for this narrative review is summarized in Figure 1. It was based on the Preferred Reporting Items for Systematic Reviews and Meta-Analyses Search Reporting Extension (PRISMA-S) guidelines [107]. It was performed by one author on 30 December 2022.

We only included articles that were published from 2002 up to and including 2022. We did not use any published search filters. Conference abstracts, letters to the editor, and case reports were excluded because they were of no use in this narrative review. With the deduplication tool in Endnote, we maximally attempted to exclude duplicate records. This deduplication was, for example, based on the title, list of authors, and year of publication. After the exclusion of the duplicates, we screened titles and abstracts for relevance. After this, we selected articles based on their content. All clinical trials, systematic reviews, and meta-analyses describing a link between CM and estrogen or estrogen receptors were eligible for this review. Other relevant articles were retrieved by checking the reference list of selected reviews. Publications where we had no free access to the full text or that were not written in English or Dutch were excluded. Other exclusion criteria include in vitro studies (except for some studies about ERs), animal studies, articles related to therapeutical implementations, articles about non-CM cancers or cancers in general, and articles about an association with obesity or fat. This led to the selection of 68 studies and 4 systematic reviews and meta-analyses.

### 4.3. Data Extraction

We extracted data based on different parameters such as the study type, sample size, study population, mean or median age of the population, number of women and men, and CM.

### 4.4. Data Analysis

The primary endpoint of this narrative review was to investigate the role of estrogen (receptors) in CM.

## 5. General Strengths and Limitations

The complexity of the topics discussed in the review posed challenges in drawing straightforward conclusions, as the existing literature did not always align. In addition, caution is warranted in interpreting these findings due to certain limitations, such as the limited sample size of some articles. Other articles did not comprehensively describe the patient population, and at the same time, descriptions of melanoma (CM) stages and the different therapeutic strategies were also often missing. Another limitation was that the prognostic value of clinical and pathological parameters, such as CM subtype or localization, was not always outlined in detail. These limitations should be taken into account when interpreting the collective findings presented in this review. A general strength, however, is that an attempt was made to provide a broad review of the literature on estrogens and CM. Our review emphasizes the need for more comprehensive research and potentially highlights areas where conflicting findings warrant further investigation.

## 6. Conclusions

**Figure 2:**
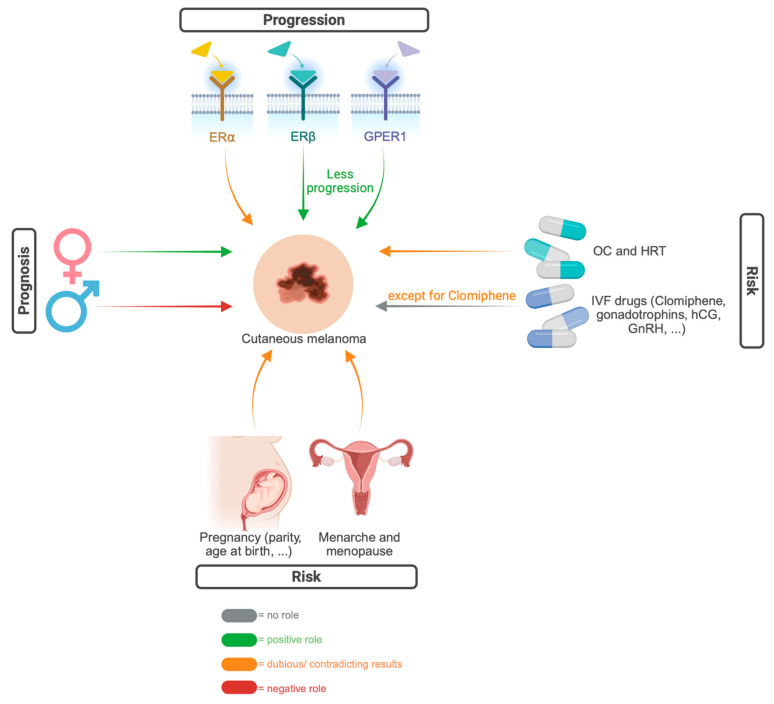
An overview of the role of estrogen (receptors) in melanoma (created with BioRender.com). Abbreviations: ER⍺ = estrogen receptor alpha, ERβ = estrogen receptor beta, GPER1 = G-protein-coupled estrogen receptor 30/1, ♀ = women, ♂ = men, OC = oral contraceptive, HRT = hormone replacement therapy, IVF = in vitro fertilization, hCG = human chorionic gonadotrophin, GnRH = gonadotrophin-releasing hormone.

Considering all the evidence presented, it is clear that estrogens and estrogen receptors play a significant role in melanoma, although there is not always unanimity on the precise relationship. We tried to provide an overview of these observations in Figure 2. The diverse findings discussed in the review collectively highlight the intricate relationship between estrogens and cutaneous melanoma, providing valuable insights for future research and potential therapeutic interventions in this context. Furthermore, we suggest a collaboration between researchers and clinicians to translate findings into clinical practice.

## Figures and Tables

**Table 1:** Estrogen (receptors): estrogen receptor alpha (ERα), estrogen receptor beta (ERβ), and others.

Autor and Publication Year	Study Design	Sample Size	Mean/ Median * Age	Number of Women/Men	Type of Biological Material	Role of ER in Melanoma (CM)
**ER** **α**						
Mori et al., 2006 [24]	Retrospective clinical study	216 CM (109 sera, 107 tumor) stage I to IV	Tumor: NA; sera: 45 *	Tumor: 49/58; sera: 34/73	Tissue and serum	↑ ERα → progression.2
Glatthaar et al., 2015 [25]	Retrospective case-control study	413 (205 CM and 208 controls)	Cases: 60–90; controls: <30	Cases: 100/105 Controls: 111/97	Blood	SNP 6362C/T variant allele: disease course < 1 year and fewer relapses. SNP −4576A/C on wild-type allele: ↓ CM risk, but ↑ chemotherapy. Cases: ↑ +1619A/G wild type. Controls: ↑ +1619A/G and +6362C/T variants.
Rajabi et al., 2017 [21]	Retrospective cross-sectional study	38 CM	52.4	20/18	Tissue	No presence of ERα in CM.
**ERβ**						
De Giorgi et al., 2013 [26]	Prospective comparative study	66 CM	NA	44/22	Tissue	↓ ERβ → progression.
Asadi et al., 2013 [27]	Retrospective case-control study	21 (11 CM, 10 nevi)	CM: 54, nevi: 43	CM: 8/3 Nevi: 4/6	Tissue	↓ ERβ → progression.
**ER** **α** **, Erβ, and others**						
Schmidt et al., 2006 [28]	Retrospective comparative study	94 (36 CM)	NA	NS	Tissue	↓ ERβ → progression. ERβ (not ERα) is predominant in CM.
Ohata et al., 2008 [20]	Prospective comparative study	40 (12 CM and 28 nevi)	CM: ♀48.5 *, ♂57 *; nevi: ♀27.2 *, ♂21.3 *	CM: 6/6 Nevi: 17/11	Tissue	No presence of ERα and ERβ is predominant in CM.
De Giorgi et al., 2009 [29]	Prospective comparative study	14 (12 CM and 2 nevi)	NA	7/7	Tissue	↓ ERβ and ERα → progression. Presence of ERα and ERβ in CM.
De Giorgi et al., 2011 [30]	Prospective clinical study	307 (112 CM and 195 controls)	Cases: 56 *; controls: 52 *	Cases: 48/64 Controls: 85/110	Blood	ERβ Alul restriction site polymorphism → ↑ risk. ERβ pp polymorphism correlates with Breslow thickness. ERα and ERβ polymorphism → progression.
Spyropoulos et al., 2013 [31]	Retrospective clinical study	60 CM	54.4	19/41	Tissue	↓ ERβ → progression. ERβ (not ERα) is predominant in CM.
Fabian et al., 2017 [32]	Retrospective comparative study	81 CM (38 P and 43 NP)	P♀: 33.07; NP♀: 33.75; ♂: 34.34	60 (38 P, 22 NP)/21	Tissue	↓ ERβ and GPER1 → progression.
Yuan et al., 2020 [33]	Retrospective clinical study	3463 (GEM: 177 CM and 172 controls; GENEVA 2054 CM and 1060 controls)	NA	GEM cases: 83/94; controls: 74/98 GENEVA cases: 867/1187; controls: 432/628	Tissue	GG phenotype in IGF1 rs1520220: men have ↑ risk, women have ↓ risk. AA genotype in IGF1R rs2229765: protective in men, no effect in women. Possible role of ESR1 SNPs rs2234693 and rs827421.
Spalkowska et al., 2021 [34]	Prospective cross-sectional study	73 (24 CM, 24 dysplastic nevi, 25 common nevi)	43 *	48/25	Tissue	↓ ERβ and GPER1 in CM. No differences in ERα expression in melanocytic lesions.
Larsson et al., 2022 [35]	Retrospective cohort study	3130 CM	NA	All♀	Serum	No role of E2 in CM.
Dika et al., 2022 [36]	Retrospective pilot study	28 CM	NA	All♀	Tissue	↑ ERβ and ERα in CM with a history of breast cancer.

Abbreviations: NA = not available, ♀ = women, ♂ = men, P = pregnant, NP = non pregnant, E2 = 17β-estradiol, ↑ = higher/more, ↓ = lower/less, → = causes. (* Indicates median age).

**Table 2:** Differences between sexes: women and men.

Autor and Publication Year	Study Design	Sample Size	Mean/Median * Age	Number of Women/Men	Role of Sex in Melanoma (CM)
**Women**					
de Vries et al., 2008 [37]	Retrospective cohort study	10,538 CM	♀53.18, ♂55.29	57.9%/52.1%	Better prognosis.
Joosse et al., 2011 [7]	Retrospective cohort study	11,774 CM stage I–IV	♀55.9, ♂57.2	5995/5779	Better prognosis.
Joosse et al., 2012 [38]	Retrospective cohort study	2672 CM stage I–II	♀50.1, ♂52.5	1398/1274	Better prognosis.
Joosse et al., 2013 [39]	Retrospective pooled analysis of five RCTs	4040 CM stage III–IV	NA	Stage III: 1162/1572 Stage IV: 541/765	Better prognosis.
Morgese et al., 2020 [40]	Retrospective clinical study	1023 CM stage I–IV	54	536/487	Better prognosis, but no difference in stages III and IV.
**Men**					
Scoggins et al., 2006 [41]	Prospective cohort study	3324 CM (≥1 mm Breslow)	18–70	1418/1906	Worse prognosis.
Gamba et al., 2013 [42]	Retrospective cohort study	26,107 CM	♀31.2, ♂31.9	15,729/10,378	Worse prognosis.
**Women and men**					
Strouse et al., 2005 [43]	Retrospective clinical study	3928 CM	Pediatric (<20 years) or young adult (20–24 years)	2069/1859	Worse prognosis in men and older age. ↓ CM incidence in men and earlier incidence in women.
Lasithiotakis et al., 2008 [44]	Retrospective cohort study	4785 CM (Clark > I no metastasis)	NA	2688/2097	Worse prognosis in men and old age (more obvious in ♀). No difference in prognosis when >65 years.
Enninga et al., 2017 [45]	Retrospective cohort study	106,511 CM stage I–IV	NA	47,687/58,824	Better prognosis of localized and regional CM in women. Equal prognosis in metastatic CM for women and men. Worse prognosis in old age (more obvious in women).
Liu et al., 2012 [46]	Retrospective comparative study	228,035 CM	NA	126 233/101 802	↑ CM incidence in women when ≤44 years and when ≥44 years ↑ in men.
Hernando et al., 2016 [47]	Retrospective case-control study	1057 (550 CM and 507 controls)	♀45 *, ♂47 *	Cases: 316/234 Controls: 283/224	↑ risk in men, possibly related to sex-specific SNPs (TYR, SILV/CDK2, GPR143, and F2RL1).
Schmidt et al., 2006 [28]	Retrospective comparative study	94 (36 CM)	NA	NS	No difference in ERβ expression between sexes.
Ohata et al., 2008 [20]	Prospective comparative study	40 (12 CM and 28 nevi)	CM: ♀48.5 *, ♂57 *; nevi: ♀27.2 *, ♂21.3 *	CM: 6/6; nevi: 17/11	No difference in ERβ expression between sexes.
De Giorgi et al., 2013 [26]	Prospective comparative study	66 CM	NA	44/22	↓ ERβ in men and postmenopausal women.
Fabian et al., 2017 [32]	Retrospective comparative study	81 CM (38 P and 43 NP)	P♀: 33.07; NP♀: 33.75; ♂: 34.34	60 (38 P, 22 NP)/21	No difference in ERβ/GPER expression between the sexes.

Abbreviations: NA = not available, RCT = randomized controlled trial, ♀ = women, ♂ = men, SNPs = single nucleotide polymorphisms, ERβ = estrogen receptor beta, GPER = G protein coupled estrogen receptor, ↑ = higher/more, ↓ = lower/less. (* Indicates median age).

**Table 3:** Oral contraceptives (OC), hormone replacement therapy (HRT), and other types of exogenous hormones.

Autor and Publication Year	Study Design	Sample Size	Mean/Median * Age	Number of Women/Men	Role of Exogenous Hormones in Melanoma (CM)
**OC**					
Vessey et al., 2006 [48]	Retrospective cohort study	17,032 (94 CM)	Between 25–39	All♀	No role.
Benyi et al., 2014 [49]	Retrospective cohort study	369 (172 E treated, 197 untreated, 4 CM)	Treated: 13.2; untreated: 13	All♀	Yes, ↑ risk with ↑ dose E.
Cervenka et al., 2018 [50]	Retrospective cohort study	79,365 (539 CM)	28.7 (start OC)	All♀	Yes, ↑ risk with ↑ dose E.
**HRT**					
Mackie et al., 2004 [51]	Prospective cohort study	206 CM (83 HRT, 123 no HRT, stage I–II)	HRT: 48.65; non-HRT: 50.52	All♀	No role.
Tang et al., 2011 [52]	Retrospective randomized controlled trial	27,347 (CM: E + P 29, E 17; placebo E + P: 28, E 21)	Between 50–79	All♀	No role.
Simin et al., 2017 [53]	Retrospective cohort study	290,186 (CM NS)	NA	All♀	Yes, ↑ risk.
Botteri et al., 2017 [54]	Retrospective cohort study	684,696 (1768 CM)	Non-HRT users: 59 *; HRT: 58 *, E: 60 *, EP: 56 *, Tibolone: 56 *	All♀	Yes, ↑ risk with ↑ dose E, not with E+P.
Cervenka et al., 2019 [55]	Retrospective cohort study	75,523 (444 CM)	52.3	All♀	Yes, moderate ↑ risk.
Hicks et al., 2019 [56]	Retrospective case-control study	Case-control: 173,859 (8279 cases, 165,580 controls); Cohort: 6575	Case-control *: 62; Cohor t*: no-HRT 62, past 59, pre-diagnose 52, continuous 62, new 52	All♀	Yes, moderate ↑ risk, not dose-dependent.
Botteri et al., 2019 [57]	Retrospective cohort study	293,570 (1695 CM)	59.2	All♀	Yes, ↑ risk with E, less with E+P, not dose-dependent.
Tang et al., 2020 [58]	SR and MA	1,164,077 (4273 CM)	55	All♀	Yes, ↑ risk with E, less with E+P.
Botteri et al., 2022 [59]	Retrospective cohort study	18,850 (356 CM)	NA	All♀	Yes, ↑ risk with E, not with E+P.
Lallas et al., 2022 [60]	SR and MA	2,612,712 (20,150 CM)	NA	All♀	Yes, ↑ risk.
**OC and HRT**					
Freedman et al., 2003 [61]	Retrospective cohort study	68,588 (207 CM)	NA	159♀, 48♂	No role.
Naldi et al., 2005 [62]	Retrospective case-control study	624 (316 CM and 308 controls)	NA	All♀	No role.
Lea et al., 2007 [63]	Retrospective case-control study	713 (318 CM and 395 controls)	NA	All♀	No role.
Gandini et al., 2011 [64]	SR and MA	OC/HRT: 5626 CM	41	All♀	No role.
Donley et al., 2019 [65]	Retrospective cohort study	167,503 (1061 CM)	CM cases: 62.6; no CM: 62.2	All♀	No role.
Koomen et al., 2009 [66]	Retrospective clinical study	687 CM	53.3	All♀	No role in Breslow thickness.
Koomen et al., 2009 [67]	Retrospective case-control study	4850 (778 CM and 4072 controls)	Cases: 53.6; Controls: 54.6	All♀	Yes, ↑ risk, dose-dependent.
De Giorgi et al., 2017 [68]	Retrospective case-control study	1197 (605 CM and 592 controls)	Cases: 60 *; Controls: 39 *	Cases: 332♀, 273♂; Controls: 337♀, 255♂	Yes, ↓ risk.
Cervenka et al., 2020 [69]	Retrospective cohort study	OC: 334,483 (1696 CM); HRT: 134,758 (770 CM)	51.1	All♀	Yes, moderate ↑ risk.
Sun et al., 2020 [70]	SR and MA	3,571,910 (CM NS)	NA	All♀	Yes, ↑ risk with HRT, OC ≥ 5 years, or 5–19 years prior.
**Other types of exogenous hormones**					
Althuis et al., 2005 [71]	Retrospective cohort study	8422 (42 CM)	30 *	All♀	No role of C or G.
Hannibal et al., 2008 [72]	Retrospective cohort study	54,362 (112 CM)	30 *	All♀	No role of C, G, hCG, or GnRH.
Silva et al., 2009 [73]	Retrospective cohort study	7355 (14 CM)	28.1	All♀	No role of C or G.
Yli-Kuha et al., 2012 [74]	Retrospective cohort study	18,350 (9175 cases, 9175 controls and 21 CM)	NA	All♀	No role of IVF drugs.
Spaan et al., 2015 [75]	Prospective cohort study	25,108 (19,158 IVF, 5950 non-IVF, 93 CM)	IVF: 49.6 *; non-IVF: 51.2 *	All♀	No role of IVF drugs.
Mai et al., 2022 [76]	Retrospective cohort study	46,544 (444 CM)	NA	All♀	No role of OC, HRT, or DES in utero.
Calderon-Margalit et al., 2009 [77]	Retrospective cohort study	15,030 (78 CM)	No treatment: 27.5; Treatment: 28.1; C: 27.9	All♀	Yes, ↑ risk with C.
Verloop et al., 2010 [78]	Prospective cohort study	12,091 (48 CM)	44 *	All♀	Yes, ↑ risk ≤40 years with DES in utero.
Stewart et al., 2013 [79]	Prospective cohort study	21,604 (149 CM)	31.2	All♀	Yes, ↑ risk with IVF in parous ♀.
Brinton et al., 2015 [80]	Retrospective cohort study	9892 (70 CM)	30.1 and 47 at CM diagnosis	All♀	Yes, ↑ risk with C.
Lai et al., 2015 [81]	Retrospective clinical study	59,205 (15,688 urinary phytoestrogen, 50 CM)	59 *	CM cases: 82♀, 90♂	↓ Tofu in diet and ↓ phytoestrogens in urine. (NSS)
Dika et al., 2017 [82]	Retrospective clinical study	28 CM	NA	All♀	Yes, ERα and PR in CM after IVF.
Dika et al., 2022 [36]	Retrospective pilot study	28 CM	NA	All♀	Yes, ↓ ERs in CM with ovarian stimulation.

Abbreviations: SR = systematic review, MA = meta-analysis, NA = not available, ♀ = women, ♂ = men, E = estrogen, P = progesterone, C = clomiphene, G = gonadotrophins, DES = diethylstilbestrol, IVF = in vitro fertilization, NSS = not statistically significant, ↑ = higher/more, ↓ = lower/less. (* Indicates median age).

**Table 4:** Different life stages.

Autor and Publication Year	Study Design	Sample Size	Mean/Median * Age	Number of Women/Men	Role of Different Life Stages in Melanoma (CM)
**Pregnancy (e.g., parity, birth, etc.)**					
Daryanani et al., 2003 [83]	Retrospective cohort study	414 CM stage I–II (46 P, 368 NP)	P: 30; NP: 36	All♀	No role.
Lens et al., 2004 [84]	Retrospective cohort study	5533 CM (185 P, 5348 NP)	P: 29.3; NP: 35	All♀	No role.
O’Meara et al., 2005 [85]	Retrospective cohort study	2863 CM (412 P, 2451 NP)	NA	All♀	No role.
Hannibal et al., 2008 [72]	Retrospective cohort study	54,362 (112 CM)	30 *	All♀	No role.
Neale et al., 2005 [86]	Retrospective cohort study	1,234,967 (2285 CM)	34	All♀	Yes, ↑ risk with older age at first birth and ↓ risk with ↑ maternity.
Karagas et al., 2006 [87]	Retrospective pooled analysis of 10 case-control studies	5590 (2391 CM, 3199 controls)	NA	All♀	Yes, ↓ risk with younger age at first birth and ↑ parity.
Kaae et al., 2007 [88]	Retrospective cohort study	3,536,175 (9596 CM)	NA	1,725,627 (5688 CM)/ 1,810,548 (3908 CM)	Yes, ↓ risk with younger age at first birth and ↑ parity. Possible role of lifestyle factors.
Gandini et al., 2011 [64]	SR and MA	Reproductive factors: 16,787 CM	41	All♀	Yes, ↑ risk with older age at first birth and ↓ risk with ↑ parity.
Spaan et al., 2015 [75]	Prospective cohort study	25,108 (19,158 IVF, 5950 non-IVF, 93 CM)	IVF: 49.6 *; non-IVF: 51.2 *	All♀	Yes, ↓ risk with younger age at first birth.
Miller et al., 2010 [89]	Retrospective comparative study	76 CM (11 P, 65 NP)	34	All♀	Yes, worse prognosis.
Zhou et al., 2014 [90]	Retrospective comparative study	54 CM (18P, 36 NP)	P: 30 *; NP♀31 *, ♂30*	36 (18 P, 18NP)/18	No role of ERβ in survival. ↑ ERβ in P women than in men, no difference between P and NP women. (NSS)
Fabian et al., 2017 [32]	Retrospective comparative study	81 CM (38 P, 43 NP)	P ♀: 33.07; NP♀: 33.75; NP♂: 34.34	60 (38 P, 22 NP)/21	No role, GPER and ↑ ERβ in CM of P women.
Hrgovic et al., 2021 [91]	Prospective comparative study	103 (78 stage I-IV CM, 10 NP controls, 15 P controls)	Cases: 61; NP controls: 41; P controls: 32	Cases: 26/52; controls: 19/6	No role of 2-ME. ↑ 2-ME in P controls.
**Menarche, menopause, and pregnancy (e.g., parity, birth, etc.)**					
Freedman et al., 2003 [61]	Retrospective cohort study	68,588 (207 CM)	NA	159/48	No role.
Naldi et al., 2005 [62]	Retrospective case-control study	624 (316 CM, 308 controls)	NA	All♀	Yes, ↑ risk with older age at first and last birth. No role for menopause or the number of births.
Lea et al., 2007 [63]	Retrospective case-control study	713 (318 CM, 395 controls)	NA	All♀	Yes, ↑ risk in livebirth 5 years before CM and number of births when <55 years old. No role of age at menarche, menopause, or the first or last pregnancy.
Kvaskoff et al., 2011 [92]	Retrospective cohort study	91,972 (460 CM)	NA	All♀	Yes, ↓ risk in women ≥15 years at menarche, <48 years at menopause, with an irregular menstrual cycle and shorter reproductive life. Modest ↓ risk with high parity. No role of age at first or last birth.
Donley et al., 2019 [65]	Retrospective cohort study	167,503 (1061 CM)	CM cases: 62.6 no CM patients: 62.2	All♀	Yes, ↑ risk with young age at menarche and older at menopause. No role for parity or age at first birth.
Sun et al., 2020 [70]	SR and MA	3,571,910 (CM NS)	NA	All ♀	Yes, ↑ risk with low parity and being ≥20 years old at first birth. No role of age in menarche and menopausal status.
Mai et al., 2022 [76]	Retrospective cohort study	46,544 (444 CM)	NA	All♀	Yes, ↑ risk with young age at menarche and older at first birth.
Joosse et al., 2012 [38]	Retrospective cohort study	2672 CM stage I–II	♀50.1, ♂52.5	1398/1274	Pre- and postmenopausal women have a better prognosis than men.
Joosse et al., 2013 [39]	Retrospective pooled analysis of five RCTs	4040 CM stage III–IV	NA	Stage III: 1162/1572; stage IV: 541/765	Pre- and postmenopausal women have a better prognosis than men.
Enninga et al., 2017 [45]	Retrospective cohort study	106,511 CM stage I–IV	NA	47 687/58,824	Pre- and perimenopausal women have a better prognosis than men.
Ohata et al., 2008 [20]	Prospective comparative study	40 (12 CM, 28 nevi)	CM: ♀48.5 *, ♂57 *; nevi: ♀27.2 *, ♂21.3 *	CM: 6/6; nevi: 17/11	No different ERβ immunostaining in different ages and P or NP.
De Giorgi et al., 2013 [26]	Prospective comparative study	66 CM	NA	44/22	↓ ERβ in the CM of postmenopausal women

Abbreviations: SR = systematic review, MA = meta-analysis, NA = not available, RCT = randomized controlled trial, ♀ = women, ♂ = men, P = pregnant, NP = non-pregnant, NSS = not statistically significant, 2-ME = 2-methoxyestradiol, ↑ = higher/more, ↓ = lower/less. (* Indicates median age).

## Data Availability

No new data were created or analyzed in this study. Data sharing is not applicable to this article.

## Abbreviations

2-ME	2-methoxyestradiol
CM	Cutaneous melanoma
DES	Diethylstilbestrol
E2	17β-estradiol
ER	Estrogen receptor
ERα	Estrogen receptor alpha
ERβ	Estrogen receptor beta
ESR1	Gene for encoding estrogen receptor alpha
ESR2	Gene for encoding estrogen receptor beta
GnRH	Gonadotrophin-releasing hormone
GPR30 or GPER1	G-protein-coupled estrogen receptor 30/1
hCG	Human chorionic gonadotrophin
HRT	Hormone replacement therapy
IGF1	Insulin-like growth factor 1
IGF1R	Insulin-like growth factor 1 receptor
IVF	In vitro fertilization
OC	Oral contraceptive
PR	Progesterone receptor
PRISMA-S	Preferred Reporting Items for Systematic Reviews and Meta-Analyses Search Reporting Extension
RCT	Randomized controlled trial
SERM	Selective estrogen receptor modulator
SNP	Single Nucleotide Polymorphism
SSM	Superficially spreading cutaneous melanoma
UV	Ultraviolet

## Appendix A

**Table A1:** Search terms in the databases PubMed, Embase, ClinicalTrials.gov, and Trialsearch.who.int.

Database	Search Terms
PubMed	Concept 1: Melanoma “Melanoma” [Mesh] OR “Melanoma*” [tiab] OR “Melanocarcinoma*” [tiab] OR “Melano-carcinoma*” [tiab] OR “Melanoma, Cutaneous Malignant” [Supplementary Concept] OR Melanomalignoma* [tiab] OR Naevocarcinoma* [tiab] OR Nevocarcinoma* [tiab] Concept 2: Estrogen receptors OR Estrogen levels “Receptors, Estrogen” [Mesh] OR “Estrogens” [Mesh] OR Estrogen* [tiab] OR Oestrogen* [tiab] OR ERalpha [tiab] OR ERbeta[tiab] OR “ER alpha” [tiab] OR “ER beta” [tiab] OR Estradiol* [tiab] Concept 3: NOT (uveal melanoma or mucosal melanoma or eye melanoma or central nervous system melanoma or esophageal melanoma or gastrointestinal melanoma or melanoma of the urethra) “Uveal melanoma” [Supplementary Concept] OR “Uveal-melanoma*” [tiab] OR “Eye-melanoma*” [tiab] OR “Mucous Membrane” [Mesh] OR Mucous-membrane* [tiab] OR Mucosa [tiab] OR “Central nervous system melanoma*” [tiab] (#1 AND #2) NOT #3
Embase	Concept 1: Melanoma ‘Melanoma’/exp OR ‘Melanoma*’:ti,ab,kw OR ‘Melanocarcinoma*’:ti,ab,kw OR ‘Melano-carcinoma*’:ti,ab,kw OR Melanomalignoma*:ti,ab,kw OR Naevocarcinoma*:ti,ab,kw OR Nevocarcinoma*:ti,ab,kw Concept 2: Estrogen receptors OR Estrogen levels ‘Estrogen receptor’/exp OR ‘Estrogen’/exp OR ‘Estrogen blood level’/exp OR Estrogen*:ti,ab,kw OR Oestrogen*:ti,ab,kw OR ERalpha:ti,ab,kw OR ERbeta:ti,ab,kw OR ‘ER alpha’:ti,ab,kw OR ‘ER beta’:ti,ab,kw OR Estradiol*:ti,ab,kw Concept 3: NOT (uveal melanoma or mucosal melanoma or eye melanoma or central nervous system melanoma or esophageal melanoma or gastrointestinal melanoma or melanoma of the urethra) ‘Eye melanoma’/exp OR ‘Central nervous system melanoma’/exp OR ‘Uveal-melanoma*’:ti,ab,kw OR ‘Eye-melanoma*’:ti,ab,kw OR ‘mucosa’/exp OR Mucous-membrane*:ti,ab,kw OR Mucosa:ti,ab,kw OR ‘Central nervous system melanoma*’:ti,ab,kw (#1 AND #2) NOT #3 NOT ‘conference abstract’:it
ClinicalTrials.gov (studies with results)	Condition or disease: Melanoma OR Melanocarcinoma OR Melanomalignoma OR Naevocarcinoma OR Nevocarcinoma Other terms: “Estrogen Receptor” OR Estrogen OR Oestrogen OR ERalpha OR ERbeta OR “ER alpha” OR “ER beta” OR Estradiol
Trialsearch.who.int (studies with results)	Concept 1: Melanoma OR Melanocarcinoma OR Melanomalignoma OR Naevocarcinoma OR Nevocarcinoma Concept 2: “Estrogen Receptor” OR Estrogen OR Oestrogen OR ERalpha OR ERbeta OR “ER alpha” OR “ER beta” OR Estradiol #1 AND #2
